# Use of admixture and association for detection of quantitative trait loci in the Type 2 Diabetes Genetic Exploration by Next-Generation Sequencing in Ethnic Samples (T2D-GENES) study

**DOI:** 10.1186/1753-6561-8-S1-S6

**Published:** 2014-06-17

**Authors:** Daniel Yorgov, Karen L Edwards, Stephanie A Santorico

**Affiliations:** 1 Department of Mathematical and Statistical Sciences, University of Colorado Denver, Denver, CO 80217-3364, USA; 2 Department of Epidemiology, School of Medicine, University of California Irvine, Irvine, CA 92697-7550, USA; 3 Department of Epidemiology, Institute for Public Health Genetics, University of Washington, Seattle, WA 98115, USA

## Abstract

Admixture mapping and association testing have been successfully applied to the detection of genes for complex diseases. Methods have also been developed to combine these approaches. As an initial step to determine the feasibility of combining admixture and association mapping in the context of whole genome sequencing, we have applied several methods to data from the Genetic Analysis Workshop 18. Here, we describe the steps necessary to carry out such a study from selection of reference populations and preprocessing of data through to the testing itself. We detected one significant result with a Bonferroni corrected p-value of 0.032 at single nucleotide polymorphism rs12639065. Computing local ancestry for Hispanic populations was challenging because there are relatively few methods by which to handle 3-way admixture, and publicly available Native American reference panels are scarce. However, combining admixture and association is a promising approach for detection of quantitative trait loci because it might be able to elevate the power of detection by combining 2 different sources of genetic signal.

## Background

Whole genome sequencing (WGS) is fast becoming a feasible technology for use in genetic studies of complex traits. Such a rich source of data allows for many methodological approaches; however, with the sheer increase in volume of data, alternatives must be evaluated with regard to their validity and power. For example, in the context of marker panels, methods for admixture mapping and association testing have been used for detection of genes for complex traits [1-3]. Combined admixture and association approaches have also been developed [4-8]. As an initial step to determine the feasibility of a combined approach in the context of WGS, we have applied several methods to data from the Genetic Analysis Workshop 18 (GAW18). We demonstrate the use of estimates of local and global ancestry in the context of an admixed population and both combine and compare ancestry information with the use of genetic association testing. Our goals are to (a) identify the steps and issues in estimation of local and global ancestry proportions for an admixed population that is best represented by more than 2 reference populations, (b) construct a series of models and test statistics that use ancestry and/or genotype data, (c) perform tests on chromosome 3 data from the GAW18 workshop for both systolic blood pressure (SBP) and diastolic blood pressure (DBP), and (d) compare the tests with respect to findings.

## Methods

### Study samples

The GAW18 data set consists of WGS data that were obtained for Mexican American families sampled from San Antonio, Texas, as a part of the Type 2 Diabetes Genetic Exploration by Next-Generation Sequencing in Ethnic Samples (T2D-GENES) consortium [9]. The genotype data were cleaned of Mendelian errors for 959 individuals, including 464 individuals who had been sequenced, with the genotypes of the remaining individuals imputed on the basis of genome-wide association data.

The ancestry estimates in this article were produced for all individuals using chromosome 3 markers. The regression analysis was performed on chromosome 3 on a set of 132 unrelated individuals with genotype, SBP, and DBP data as measured at their first examination. Simulated Q1 traits not influenced by individual genotypes were used for type I error rate assessment.

### Ancestry estimation

Local ancestry is defined as the number of copies of chromosomes inherited from a parental population at a given genomic location, resulting in a mosaic of segments of distinct ancestry across the chromosome and, equivalently, ancestry switches between those segments [6]. Historically, methods for local ancestry estimation have used coarse marker maps with much work done on constructing ancestry-informative marker (AIM) panels with a size of a few thousand markers over the whole genome. AIM panels typically incorporate markers with large frequency differences between the ancestral populations and minimal linkage disequilibrium (LD) between the AIMs; however, it has been suggested that using a denser set of markers naturally provides more information for local ancestry estimation [10].

Several newer methods allow for higher density marker panels and background LD, but most do not readily allow for multiple-way admixture. The method that we used, LAMP-LD, combines window-based processing within a hierarchical Hidden Markov Model and takes as an input the genotypes of the admixed individuals as well as phased haplotype reference panels representative of the ancestral populations [11]. We produced the most likely pair of local ancestries at each marker with LAMP-LD software release 1.0. Global ancestry proportions were estimated by averaging local ancestry estimates over all chromosome 3 markers used.

### Statistical models

Multiple linear regression models were fit to individual log-transformed blood pressure measurements with global ancestry proportions of European, Native American, and African descent as explanatory variables. Additional covariates were selected based on forward stepwise selection with a 0.05 significance level, resulting in log(SBP) with global ancestry, age, and blood pressure medication used as explanatory variables and log(DBP) with global ancestry and blood pressure medication used as explanatory variables. We assumed an additive genetic model using counts of minor alleles, *g*, with *0 ≤ g ≤ 2*. Because of imputation, *g *is not always an integer. For each marker, local ancestry was coded into dummy variables representing each unique combination of ancestry: *D_EE_, D_AA_, D_NN_, D_EA_, D_EN_*, and *D_NA _*where *E, N*, and *A *represent European, Native American, and African ancestry, respectively, for the allele. Four tests were conducted over chromosome 3 for the logarithm of each of the 2 blood pressure measurements, denoted by *y*, based on the following models:

***Model 1 (null): ***y = α + X'**β **+ ε

***Model 2 (association): ***y = α + X'**β **+ β_g_g + ε

***Model 3 (admixture): ***y = α + X'**β **+ [β_AA_D_AA _+ β_NN_D_NN _+ β_EA_D_EA _+ β_EN_D_EN _+ β_NA_D_NA_] + ε

*Model 4 (heterogeneous association and admixture):*

y = α + X'**β **+ [β_AA_D_AA _+ β_NN_D_NN _+ β_EA_D_EA _+ β_EN_D_EN _+ β_NA_D_NA_]    + β_g,EE_(g × D_EE_) + β_g,AA_(g × D_AA_) + β _g,NN_(g × D_NN_) + β _g,EA_(g × D_EA_) + β _g,EN_(g × D_EN_) + β _g, NA_(g × D_NA_) + ε. Here, *X *represents baseline covariates. Both Wald and likelihood ratio tests were used to test for admixture (model 3 vs. model 1), association (model 2 vs. model 1), association adjusted for admixture (model 4 vs. model 3), and admixture and/or association (model 4 vs. model 1). Wald and likelihood ratio test statistics (LRTS) are asymptotically chi-squared distributed under the standard assumption of a normally distributed error, *ε*. Local ancestry and genotype at a marker are not independent; hence, genetic effects in the full model (model 4) were stratified by individual local ancestry [6]. This also allows for heterogeneous genetic association.

To adjust for multiple comparisons, 3 permutation techniques were used. First, an individual's phenotype, global ancestry, and covariates were randomized relative to his or her genotype and local ancestry vectors. Empirical significance thresholds for a family-wise error rate (FWER) of 0.05 were computed based on 1000 permutations. Second, for a marker significant for one of the tests based on the FWER of 0.05, p-values were estimated based on more than 5 × 10^7 ^permutations. Last, to assess type I error rates, the admixture and/or association statistics were computed on all markers used for 200 simulated data sets for a trait not influenced by the genotype (Q1), including sex and age as covariates.

In a follow-up analysis for a single marker, the model below was used to explicitly test for heterogeneity of the genetic association effect by comparison with model 4:

*Model 5 (homogenous association and admixture):*

y = α + X'**β **+ [β_AA_D_AA _+ β_NN_D_NN _+ β_EA_D_EA _+ β_EN_D_EN _+ β_NA_D_NA_] + β_g_g + ε

### Reference panels for local ancestry estimation

The current trend toward studying local admixture focuses on continental origin as opposed to finer scale identification to region of origin within a continent. It is widely accepted that modern Hispanic populations, such as the GAW18 population, are the result of recent admixture of 3 continental-level ancestral populations, namely European, Native American, and African [12,13].

HapMap phase 3 release 2 genotypes for 112 unrelated CEU (Utah residents with ancestry from Northern and Western Europe) and 113 unrelated YRI (Yoruba in Ibadan, Nigeria) individuals were used as proxy reference panels for European and African ancestral components, respectively [14]. For the Native American reference panel, a subset of 64 individuals with at most third-degree relatedness was obtained from the Human Genome Diversity Project (HGDP) Native American populations (Colombian, Karitiana, Maya, Pima, and Surui) [15,16]. Single-nucleotide polymorphisms (SNPs) with greater than 0.2 missingness per marker were removed.

To avoid possible bias as a result of different pipelines and phasing methods, HGDP and HapMap data sets were phased using the segmented haplotype estimation and imputation tool (SHAPEIT) method [17].

### Data processing and merging

Whereas HapMap and HGDP reference data sets are based on NCBI Build 36.3 genomic coordinates, the GAW18 data set uses Build 37.3 coordinates. To allow for full confidence in the mapping between the 2, only markers in the genome-wide data set with available rs numbers in the VCF GAW18 data files were used. Chromosome 3 markers present in the 3 data sets were extracted. This resulted in a set of 40098 SNPs (denoted by SNP40098) with an average intermarker distance of 4932 bp.

### Local ancestry estimation considerations

Johnson *et al *give estimated average global ancestry proportions for the HapMap Mexican sample in Los Angeles (MXL) and for a cohort of 492 parent-offspring trios recruited from Mexico City (MEX1) [13]. Those proportions are 49% European, 45% Native American, and 5% African for the MXL panel and 31% European, 65% Native American, and 3% African for the MEX1 panel.

Assuming a 2-way admixed population, an estimate for the number of ancestry switches in a diploid genome is given by the formula *B = (2 × 2 × 0.01) TLz(1-z) *where *T *is generations since admixture, *L *is the total chromosome length (224.6cM for chromosome 3 for the genetic map used), and *z *is global proportion for one of the ancestral components [13]. The same authors estimated 10 to 15 generations since admixture for Hispanic populations.

Besides SNP40098, 2 smaller subsets constructed by selecting AIMs were also used. First, marker information content for ancestry using the f value for all 40098 markers between each pair of the 3 reference populations [18] was estimated. Based on the f value, 2 additional marker sets were constructed. For the SNP6884 marker set, which includes 6884 SNPs, all SNPs that have f >0.25 in at least one of the 3 comparisons between the reference populations were included. For the SNP637 marker set, which includes 637 markers, all SNPs in SNP40098 that have f > 0.25 between both the CEU-HGDP and YRI-HGDP reference populations were included. By construction, SNP637 is a proper subset of SNP6884, which is a proper subset of SNP40098.

## Results and discussion

We first present results of ancestry estimation. Then we provide findings from the statistical tests proposed. Last, we interpret the regression model at a marker found to have significant association and/or admixture.

### Ancestry estimation

Assuming 12 generations since admixture and *z = 0.49*, we estimated *B = 26.9 *average number of ancestry switches and 27.9 average ancestry blocks in chromosome 3 for the GAW18 population. We used this result together with the global ancestry proportions estimated for MXL to evaluate different parameters for the LAMP-LD method and different SNP subsets on which to base the local ancestry estimation.

Ancestry estimates produced with LAMP-LD are summarized in Table 1. This software allows for 2 parameters: window size in number of SNPs and number of founders in the virtual reference populations for the implemented Markov chain [11]. Different values for the second parameter gave comparable global ancestry estimates; the runs reported were based on 25 founders. The window-size parameter should depend on the resolution of the marker set used. All LAMP-LD runs were stable in terms of global ancestry proportion estimates produced apart for the run on windows of size 2. Both SNP6884 and SNP637 marker sets produced unsatisfactory results with respect to the number of ancestry switches estimated. It seems that the software is optimized for marker densities similar to the SNP40098 set [11]. In particular, the padding around a window of any size seems to be fixed, which might be the reason for the lower number of ancestry switches produced by all runs with the smaller sets.

**Table 1:** LAMP-LD ancestry estimates for different marker sets and parameters

Marker set	Window size (no. of SNPs)	Average global ancestral proportions			Number of ancestry switches	
			
		European	Native American	African	Mean	Standard deviation
SNP40098	50	0.489	0.455	0.057	26.71	6.47
***SNP40098***	***70***	***0.491***	***0.453***	***0.056***	***25.51***	***6.06***
SNP40098	100	0.491	0.453	0.056	24.76	5.86
SNP6884	5	0.486	0.459	0.055	14.61	3.63
SNP6884	10	0.494	0.454	0.052	16.55	4.10
SNP637	2	0.430	0.458	0.112	5.02	1.84
SNP637	10	0.497	0.447	0.057	8.82	2.50

All estimates are based on 959 individuals using chromosome 3 markers. Bold italic type denotes ancestry estimates that were used in the subsequent linear regression models.

*SNP*, single-nucleotide polymorphism.

Local ancestry estimates based on the LAMP-LD run for the SNP40098 marker set with window size 70 were used in the subsequent linear regression models. This run produced close to the desired number of ancestry switches and global ancestry proportions. The slightly higher average African ancestry can be explained by 2 individuals with close to 50% estimated African global ancestry (51% and 53%). In summary, there were 2462 unique local ancestry vectors for the sample of 132 unrelated individuals.

The LAMP-LD method used ignores family structure; however, using all family members for estimation of local ancestry may improve the estimates because LAMP-LD builds virtual reference populations at the training phase upon which it produces its estimates. As validation, local ancestry was estimated with the set of unrelated individuals only. For a marker (rs12639065) found to have significant association and/or admixture, the resulting ancestry vector was identical to that of the full analysis.

### Statistical tests

Permutation-based significance thresholds were computed for a FWER of 0.05. Using these thresholds, no significant results were found for the test of admixture, test of association, and test of association adjusting for admixture; however, one SNP remained significant for the combined test of admixture and/or association for the log(DBP) trait. All models, fit under both null and alternative hypotheses included effects for global ancestry proportions and the selected covariates. Table 2 summarizes the p-values for all tests at this SNP location, rs12639065.

**Table 2:** Wald and likelihood ratio p-values at the significant marker for log(DBP)

Marker coordinates (Build 37.3)	Test statistic	Admixture		Association		Association adjusting for admixture		Admixture and/or association	
					
		P-value *(α-threshold)*	df	P-value *(α-threshold)*	df	P-value *(α-threshold)*	df	P-value ***(α-threshold)***	df
14390507	LRTS	2.237 × 10^-4^ *(6.107 × 10^-5^)*	4	5.878 × 10^-3^ *(1.008 × 10^-6^)*	1	6.597 × 10^-5^ *(4.585 × 10^-7^)*	5	**2.118 × 10^-7 ^***(6.883 × 10^-7^)*	9
	Wald	3.843 × 10^-4^ *(1.177 × 10^-4^)*	4	7.035 × 10^-3^ *(1.711 × 10^-6^)*	1	1.909 × 10^-4^ *(2.291 × 10^-6^)*	5	**9.974 × 10^-7 ^***(2.685 × 10^-6^)*	9

The α-thresholds are permutation derived p-values required for achieving significance at a family-wise error rate of 0.05. P-values in bold are below the respective thresholds.

*LRTS*, likelihood ratio test statistics.

The SNP is located in an intergenic region between the *LSM3 *and *SLC6A *genes. The minor allele frequency at this marker is 0.364; a Pearson chi-square goodness-of-fit test for departure from Hardy-Weinberg equilibrium gave a p-value of 0.190. Testing the residuals for the full model, the Shapiro-Wilk test for departure of normality gave a p-value of 0.479. As expected, local ancestry estimates do not change for any individual in the sample for a region around the significant marker, from 14,317,580 bp to 14,513,695 bp (the marker itself is at position 14,390,507 bp).

LRTS and Wald tests resulted in the same rank for the admixture and/or association test, producing a permutation p-value of 8.068 × 10^-7^, which corresponds to a Bonferroni adjusted p-value of 0.032 (*N *= 40,098). The permutation-estimated p-value is closer to the Wald-based p-value of 9.974 × 10^-7 ^and greater than the LRTS- produced p-value of 2.118 × 10^-7^. This is in line with our observations from the permutation threshold runs, where the LRTS produced smaller p-values and a not entirely uniform distribution but the Wald-based permutation p-values were uniform as expected under the null (data not shown).

To further evaluate the FWER, the admixture and/or association statistics were computed on all markers in SNP40098 for all 200 simulated data sets for the Q1 trait. Sex and age were used as covariates and the minimum p-values for each data set were retained. This resulted in estimated FWERs of 0.05 and 0.04 compared with the thresholds derived for the LRTS and Wald test statistic, respectively (Table 2).

### Heterogeneous association and/or admixture model at rs12639065

Linear regression parameter estimates for model 4 for the log(DBP) trait at rs12639065 are presented in Table 3. All parameter estimates for the indicators for local ancestry with at least one Native American allele are similar in magnitude (0.138, 0.145, and 0.131 for *D_EN_, D_NN_*, and *D_NA_*, respectively). Adjusting for the genotype at the marker, a Native American local ancestry at this region is related to a 15% higher DBP on average (*e*^0.14 ^≈1.15). Two of these parameters are significant (p-values <0.004), and although the parameter estimate for the indicator for Native American and African local ancestry, *D_NA_*, is not significant (p-value = 0.132), this is likely a result of the small NA sample size.

**Table 3:** Parameter estimates for model 4 at the significant marker rs12639065 for log(DBP)

Factors	Parameter estimate	Standard error	P-value (Pr >|t|)	*N* ^1^
Intercept	4.162	0.047	**<2 × 10^-16^**	
Proportion Native American (NA) Global Ancestry	-0.099	0.063	0.116	
Proportion African Global Ancestry	-0.325	0.024	0.118	
Indicator for blood pressure medication use	0.124	0.024	**1.32 × 10^-6^**	
D_EN _Indicator for European and NA local ancestry (LA)	0.138	0.047	**0.004**	67
D_EA _European and African LA	-0.053	0.083	0.526	5
D_NN _Native American LA	0.145	0.049	**0.004**	26
D_NA _Native American and African LA	0.131	0.086	0.132	4
g × D_EE _Stratified genotype: European LA	0.123	0.030	**9.06 × 10^-5^**	30
g × D_EN _Stratified genotype: European and NA LA	0.049	0.022	**0.026**	67
g × D_EA _Stratified genotype: European and African LA	-0.039	0.103	0.707	5
g × D_NN _Stratified genotype: Native American LA	-0.069	0.037	0.064	26
g × D_NA _Stratified genotype: Native American and African LA	-0.122	0.109	0.264	4

Bold type indicates p-values less than 0.05.

^1^Sample size after stratifying for local ancestry at the marker, e.g., 67 of the unrelated individuals had European and Native American ancestral alleles at rs12639065.

Although the model suggests that for entirely European local ancestry at this region, DBP is expected to be lower compared with other local ancestries, a minor allele at the marker seems to have a positive effect in such a case, with DBP expected to increase by 13% per minor allele carried at the SNP (*e*^0.123 ^≈1.13), and is also highly significant, with a p-value of 9 × 10^-5^. Also significant is the parameter estimate for the genotype stratified on Native American and European local ancestry at the region; DBP is expected to increase by 5% per minor allele carried at the marker (*e*^0.049 ^≈1.05). Interestingly, although marginally nonsignificant with a p-value of 0.064, given a Native American local ancestry at the region, the minor allele has a negative effect for the trait (DBP is estimated to be 7% lower per minor allele carried at the marker with *e*^-0.069 ^≈0.93). All parameters for stratified genotype effects with an African local ancestral component were nonsignificant, possibly because of small sample sizes.

To further illustrate the model 4 fit for log(DBP) presented in Table 3, a person with average ancestral proportions, no medication use, and no minor alleles at the marker is expected to have a DBP of 60.3 if the local ancestry at the region is entirely European and a DBP of 69.7 if the local ancestry is entirely Native American. This compares with expected DBP levels of 77.1 and 60.7 for a person with 2 minor alleles at the marker and entirely European or entirely Native American local ancestry at the region, respectively. The test for genetic heterogeneity (model 5 vs. model 4) gave an LRTS p-value of 5.39 × 10^-4^, which suggests that heterogeneity exists among the genetic association effects. The association effect acts in different directions given different ancestry in the region.

## Conclusions

Combining admixture and association information is a promising approach for detection of quantitative trait loci. Local ancestry estimation must be performed before such combined tests are conducted; however, producing quality local ancestry estimates is challenging in a multiway admixed population such as the one used in GAW18. To the best of our knowledge, all proposed methods use some form of reference panels, which serve as proxies for the ancestral populations in the admixture. Although good reference panels exist for many populations, we found that obtaining such a proxy for the Native American ancestral component present in Hispanic populations is particularly difficult. Furthermore, using 3 different data sources increases the complexity of the process because it necessarily involves aligning and intersecting the sets used. We did find the use of benchmarks to be helpful in evaluating the ancestry estimates produced. Possible benchmarks for assessing quality are global ancestry proportions for similar populations as well as model-based estimates for the expected number of ancestral blocks in a chromosome. We did not use the family structure in our local ancestry estimates. Methods for local ancestry estimation that exploit the family structure should improve on the quality of the estimates.

Although no significant results were detected for tests of admixture, association, and association adjusted for admixture, we did find a significant marker from a combined admixture and/or association test. This may indicate increased power of quantitative trait loci detection when aggregating 2 different sources of genetic signal. Simulation studies are necessary to evaluate the power of a combined approach; however, it seems that the results from our analysis demonstrate promise and the need for further studies of such a method.

Finally, the regression model at the significant SNP suggests that the genetic signal at the SNP does not act in the same direction for different local ancestral backgrounds. More work is needed to investigate the source of heterogeneity in the association effect. Although it is tempting to think of potential sources of heterogeneity as a result of ancestry or environment, another possible explanation may relate to differences in LD between the ancestral populations. A more extensive study would be needed to evaluate such differences and their effects.

## Competing interests

The authors declare that they have no competing interests.

## Authors' contributions

SAS, KLE, and DY designed the overall study, and DY and SAS conducted statistical analyses and drafted the manuscript. All authors read and approved the final manuscript.
